# p38δ controls Mitogen- and Stress-activated Kinase-1 (MSK1) function in response to toll-like receptor activation in macrophages

**DOI:** 10.3389/fcell.2023.1083033

**Published:** 2023-02-09

**Authors:** Ester Díaz-Mora, Diego González-Romero, Marta Meireles-da-Silva, Juan José Sanz-Ezquerro, Ana Cuenda

**Affiliations:** ^1^ Department of Immunology and Oncology, Centro Nacional de Biotecnología/CSIC (CNB-CSIC), Madrid, Spain; ^2^ Department of Molecular and Cellular Biology, Centro Nacional de Biotecnología/CSIC (CNB-CSIC), Madrid, Spain

**Keywords:** p38δ/p38γ, MSK1, macrophages, phosphorylation, MAPK

## Abstract

Mitogen- and Stress-activated Kinase (MSK) 1 is a nuclear protein, activated by p38α Mitogen-Activated Kinase (MAPK) and extracellular signal-regulated kinase (ERK1/2), that modulate the production of certain cytokines in macrophages. Using knockout cells and specific kinase inhibitors, we show that, besides p38α and ERK1/2, another p38MAPK, p38δ, mediates MSK phosphorylation and activation, in LPS-stimulated macrophages. Additionally, recombinant MSK1 was phosphorylated and activated by recombinant p38δ, to the same extent than by p38α, in *in vitro* experiments. Moreover, the phosphorylation of the transcription factors CREB and ATF1, that are MSK physiological substrates, and the expression of the CREB-dependent gene encoding DUSP1, were impaired in p38δ-deficient macrophages. Also, the transcription of IL-1Ra mRNA, that is MSK-dependent, was reduced. Our results indicate that MSK activation can be one possible mechanism by which p38δ regulates the production of a variety of inflammatory molecules involved in immune innate response.

## Introduction

Toll-like receptor (TLR) signalling is fundamental in the recognition of pathogen-associated pattern molecules in innate immune cells. TLR activation will trigger the secretion of inflammatory cytokines and other pro-inflammatory mediators, which function is to eliminate infectious pathogens (Kawai and Akira, 2007; Lee and Kim, 2007). The production of these pro-inflammatory mediators is controlled by the activation of several signalling pathways, including the p38 Mitogen-Activated Kinases (p38MAPKs) (Risco et al., 2012; Cuenda and Sanz-Ezquerro, 2017; Alsina-Beauchamp et al., 2018).

p38MAPKs encompasses an important group of kinases that belong to the MAPK family, together with Extracellular signal-Regulated Kinase 1/2 (ERK1/2), c-Jun N-terminal Kinases (JNKs) and ERK5. There are four p38MAPKs, p38α, p38β, p38γ and p38δ, encoded by different genes (Cuenda and Sanz-Ezquerro, 2017). p38γ and p38δ, also known as alternative p38MAPKs, are closely related kinases and differ from p38α and p38β in their substrate specificity and sensitivity to certain kinase inhibitors (Cuenda and Sanz-Ezquerro, 2017). p38γ and p38δ (p38γ/p38δ) play important roles in innate immune response and in inflammation by regulating cytokine production in myeloid cells, B and T cell activation and proliferation, as well as inflammasome activation or neutrophil migration (Risco et al., 2012; Criado et al., 2014; Escós et al., 2016; Cuenda and Sanz-Ezquerro, 2017; Alsina-Beauchamp et al., 2018). However, the specific roles and molecular mechanisms of these kinases in the innate immune responses have not been fully characterized. Recent studies have shown that p38γ/p38δ control the levels of Tumour Progression locus 2 (TPL2), the key MAP3K upstream of ERK1/2 in myeloid cells, by regulating TPL2 mRNA translation (Escós et al., 2022). This is a mechanism by which p38γ/p38δ modulate innate immune responses, since the activation of the TPL2-ERK1/2 pathway is involved in the production of several key cytokines, including Tumour Necrosis Factor α (TNFα) and Interleukin-1β (IL-1β), in response to TLR activation (Risco et al., 2012). Non-etheless, there are also evidence of a TPL2-independent role of p38γ/p38δ in the immune response (Cuenda and Sanz-Ezquerro, 2017; Alsina-Beauchamp et al., 2018).

Here we analysed how the lack of p38γ and/or p38δ affects the activation of different signalling pathways in macrophages stimulated with the TLR4 ligand, the endotoxin lipopolysaccharide (LPS), and report that the activation of Mitogen- and Stress-activated Kinase-1 (MSK1) is impaired in p38δ- and p38γ/p38δ-null cells. MSK1, and the related MSK2, are nuclear kinases activated downstream of p38α and ERK1/2 that phosphorylate the transcription factor CREB (Deak et al., 1998). MSK1 and MSK2 are functionally redundant in cells (Wiggin et al., 2002). It has been shown that the complete blockade of MSKs activation requires the simultaneous inhibition of p38α and ERK1/2 in response to LPS (McCoy et al., 2005; Ananieva et al., 2008). Also, MSKs are involved in inflammation by modulating the production of cytokines such as TNFα, IL-6, IL-12 or IL-10 (Ananieva et al., 2008; Kim et al., 2008; Darragh et al., 2010; MacKenzie et al., 2013). In this work we show that p38δ phosphorylates and activates MSK1 *in vitro*. We found that in macrophages p38δ is involved in MSK1 phosphorylation and activation, and as a result, in the phosphorylation of the transcription factor CREB and in the transcriptional induction of CREB-dependent immediate early genes such as the dual-specificity phosphatase 1 (DUSP1) or the IL-1 receptor antagonist (IL-1Ra). All these data suggest that p38δ regulates the production of different anti-inflammatory molecules by controlling the activation of the MSK-CREB axis and plays an important role in macrophages during the innate immune and inflammatory response.

## Methods

### Antibodies and kinase inhibitors

Antibodies against total ERK1/2 (#9102), phospho-ERK1/2 (Thr202/Tyr204; #9101), total IκBα (#9242), total JNK1/2 (#9252), phospho-p38MAPK (Thr180-Tyr182; #9211), total MK2 (#3042), phospho-MK2 (Thr334; #3042), total MSK1 (#3489), phospho-MSK1 (Thr581; #9595), phospho-c-Jun (Ser73; #9164) and phospho-IKKα/β (Ser176/180; #2697) were purchased from Cell Signaling Technology. Antibodies to Phospho-CREB (Ser133, #06-519) was from Millipore, anti-p38α (#sc-535) and anti-DUSP1 (#sc-373841) were from Santa Cruz, anti-active phospho-JNK1/2 (Thr183-Tyr185; #MAB1205) from R&D System, and anti-α-Tubulin (#T9026) from Sigma. Secondary antibodies from Invitrogen (Waltham, Massachusetts, United States) included Alexa Fluor 680 donkey α-sheep IgG (H + L) (#A21102), Alexa Fluor 680 goat α-rabbit IgG (H + L) (#A21109), Alexa Fluor 700 goat α-mouse IgG (H + L) (#A21036).

Kinase inhibitors SB203580 (inhibits p38α/p38β (Kuma et al., 2005)) was purchased from Selleckchem, and SB747651A (inhibits MSK1, (Naqvi et al., 2012)) from Axon Medchem. PD184352 (MKK1 inhibitor, (Kuma et al., 2005)) and BIRB0796 (p38α/p38β/p38γ and p38δ inhibitor, (Kuma et al., 2005)) were from the Division of Signal Transduction Therapy (DSTT); University of Dundee (Dundee, UK). JNK-IN-8 (JNK inhibitor (Zhang et al., 2012)) was from Calbiochem.

### Protein expression and plasmids

Activated GST-p38α, -p38β, -p38γ and p38δ were obtained from the Division of Signal Transduction Therapy (DSTT); University of Dundee (Dundee, UK) (https://mrcppureagents.dundee.ac.uk). pGEX4T-p38γD171A (GST-p38γKD), pGEX4T-p38δD168A (GST-p38δKD), pGEX4T-p38δ (GST-p38δ), pGEX6P-MSK1(GST-MSK1) and pGEX6P-CREB (GST-CREB) were from the DSTT, expressed in *E. coli* strain BL21 and purified as described in (Knebel et al., 2001).

### Animals

All mice were housed in specific pathogen‐free conditions in the CNB‐CSIC animal house. Animal procedures were performed in accordance with national and EU guidelines, with the approval of the Centro Nacional de Biotecnología Animal Ethics Committee, CSIC and Comunidad de Madrid (Reference: PROEX 316/15 and PROEX 071/19). Adult mice 12-week-old C57BL/6J-WT, p38γ/δ−/−, -p38γ−/− and -p38δ−/− were used in this work.

### Bone marrow derived macrophages culture and stimulation

Bone marrow derived macrophages (BMDM) lacking p38γ, p38δ or p38γ/δ were obtained from adult mouse femur and tibia as described elsewhere (Risco et al., 2012; Alsina-Beauchamp et al., 2018). Briefly, bone marrow cells were differentiated for 6 days on bacteria-grade plastic dishes in DMEM with 20% FBS and 30% L929 cell-conditioned media. Adherent cells were collected and plated (0.5 × 106 cells/plate) in DMEM with 0.05% FBS. After 12 h, BMDMs were stimulated in 0.1%–1% serum with 100 ng/mL LPS (Sigma-Aldrich) or with 250 ng/mL unmethylated CpG oligonucleotide (CpG-ODN, ODN-1668) (InvivoGen). Murine macrophage Raw 264.7 cells were cultured in DMEM with 10% FBS, Penicillin (100 U/mL), Streptomycin (100 μg/mL) and L-glutamine (2 mM), and stimulated with 100 ng/mL LPS. When indicated, cells were pre-treated for 1 h with DMSO, SB203580, BIRB0796, PD184352 or SB747651A. Cells were lysed in lysis buffer ([50 mM Tris-HCl (pH 7.5), 1 mM EGTA, 1 mM EDTA, 50 mM sodium fluoride, 10 mM sodium β-glycerophosphate, 5 mM pyrophosphate, 0.27 M sucrose, 1% (vol/vol) Triton X-100] plus 0.1% (vol/vol) 2-mercaptoethanol, 0.1 mM phenylmethylsulfonyl fluoride, 1 mM benzamidine and 1 mM sodium orthovanadate). Lysates were centrifuged at 20,800 g for 15 min at 4°C, the supernatants removed, quick frozen in liquid nitrogen and stored at −80°C until used.

For mRNA expression analysis, BMDM were lysed with NZYol (NZYtech) and the RNA extracted using a standard protocol with chloroform-isopropanol-ethanol.

### *In vitro* MSK1 phosphorylation

p38MAPKs were assayed using Myelin Basic Protein (MBP) as substrate (Cuenda et al., 1997). Briefly, kinase assay were set in a 30 µL final phosphorylation reaction mixture containing MBP (0.33 mg/mL), active p38MAPK (0.5 U/mL) and 50 mM Tris-HCl pH 7.5, 0.1 mM EGTA, 10 mM MgCl2 and 0.1 mM [γ32P]ATP (Amersham; specific activity: ∼3 × 106 cpms). The reactions were carried out at 30°C for 60 min and stopped by spotting the phosphorylation reaction mixture onto P81 filtermats, washed four times in 75 mM phosphoric acid to remove ATP, washed once in acetone, and then dried and counted for radioactivity incorporated into MBP. To study the MSK1 phosphorylation by active p38MAPKs, these kinases were matched for activity against MBP. Each p38MAPK (0.5 U/mL) was incubated for 60 min or the indicated times at 30°C with Mg [γ32P]ATP (specific activity: ∼3 × 106 cpms) or Mg-ATP plus 1 µM GST-MSK1. The samples were denatured by adding 4 x SDS-PAGE sample buffer containing 1% (v/v) 2-mercaptoethanol, electrophoresed and autoradiographed. Phosphorylated MSK1 was quantified using the Fiji program.

### *In vitro* MSK1 activity

MSK1 (2 μg, 0.9 µM) was first activated with p38α or p38δ (0.5 U/mL) in kinase assay buffer (50 mM Tris-HCl pH 7.5, 0.1 mM EGTA and 10 mM MgCl2) and 0.1 mM ATP. After the indicated times at 30°C, fractions containing active MSK1 were diluted 1:100 in kinase assay buffer plus 0.1 mM [γ32P]ATP (specific activity: ∼3 × 106 cpms) or 0.1 mM ATP, and GST-CREB (1 μg, 0.74 µM). The reactions were carried out at 30°C for 15 min and terminated by adding 4 x SDS-PAGE sample buffer containing 1% (v/v) 2-mercaptoethanol. Reaction samples were electrophoresed and autoradiographed. Phosphorylated MSK1 and CREB were quantified using the Fiji program.

### Immunoblotting

Protein samples were resolved in sodium dodecyl sulphate-polyacrylamide gel electrophoresis (SDS-PAGE) and transferred to nitrocellulose membranes, blocked (30 min at 25°C) in 50 mM Tris/HCl pH 7.5, 0.15 M NaCl and 0.05% (v/v) Tween (TBST buffer) with 10% (w/v) dry milk. Then membranes were incubated in TBST buffer with 5% (w/v) dry milk and 0.5–1 μg/mL antibody (2 h at 25°C or overnight at 4°C). Proteins were detected using fluorescently labelled secondary antibodies and the Odyssey infrared Imaging System (LI-COR Biosciences, Lincoln, Nebraska, United States).

### Analysis of gene expression

cDNA for real-time quantitative PCR (qPCR) was generated from total RNA using the High Capacity cDNA Reverse Transcription Kit (Applied Biosystems). Real-time qPCR reactions were performed in triplicate as described (Risco et al., 2012; Alsina-Beauchamp et al., 2018) in MicroAmp Optical 384-well plates (Applied Biosystems). PCR reactions were carried out in an ABI PRISM 7900HT (Applied Biosystems) and SDS v2.2 software was used to analyse results by the Comparative Ct Method (ΔΔCt). X-fold change in mRNA expression was quantified relative to non-stimulated wild-type cells, and β-actin mRNA was used as control. Primers used were:

IL-1Ra:

forward 5′-GGC​AGT​GGA​AGA​CCT​TGT​GT and

revers 5′-CAT​CTT​GCA​GGG​TCT​TTT​CC;

β-actin:

forward 5′-AAG​GAG​ATT​ACT​TGC​TCT​GGC​TCC​T and

revers 5′-ACT​CAT​CGT​ACT​CCT​GCT​TGC​TGA​T;

DUSP1:

forward 5′-TGG​GAG​CTG​GTC​CTT​ATT​TAT​T and

revers 5′-GAC​TGC​TTA​GGA​ACT​CAG​TGG​AA.

### Statistical analysis

Data were processed using Student’s t-test. In all cases, *p* values < 0.05 were considered significant. Data are shown as mean ± SEM.

## Results

### Lack of p38δ impairs MSK1 activation in bone marrow derived macrophages

**FIGURE 1:**
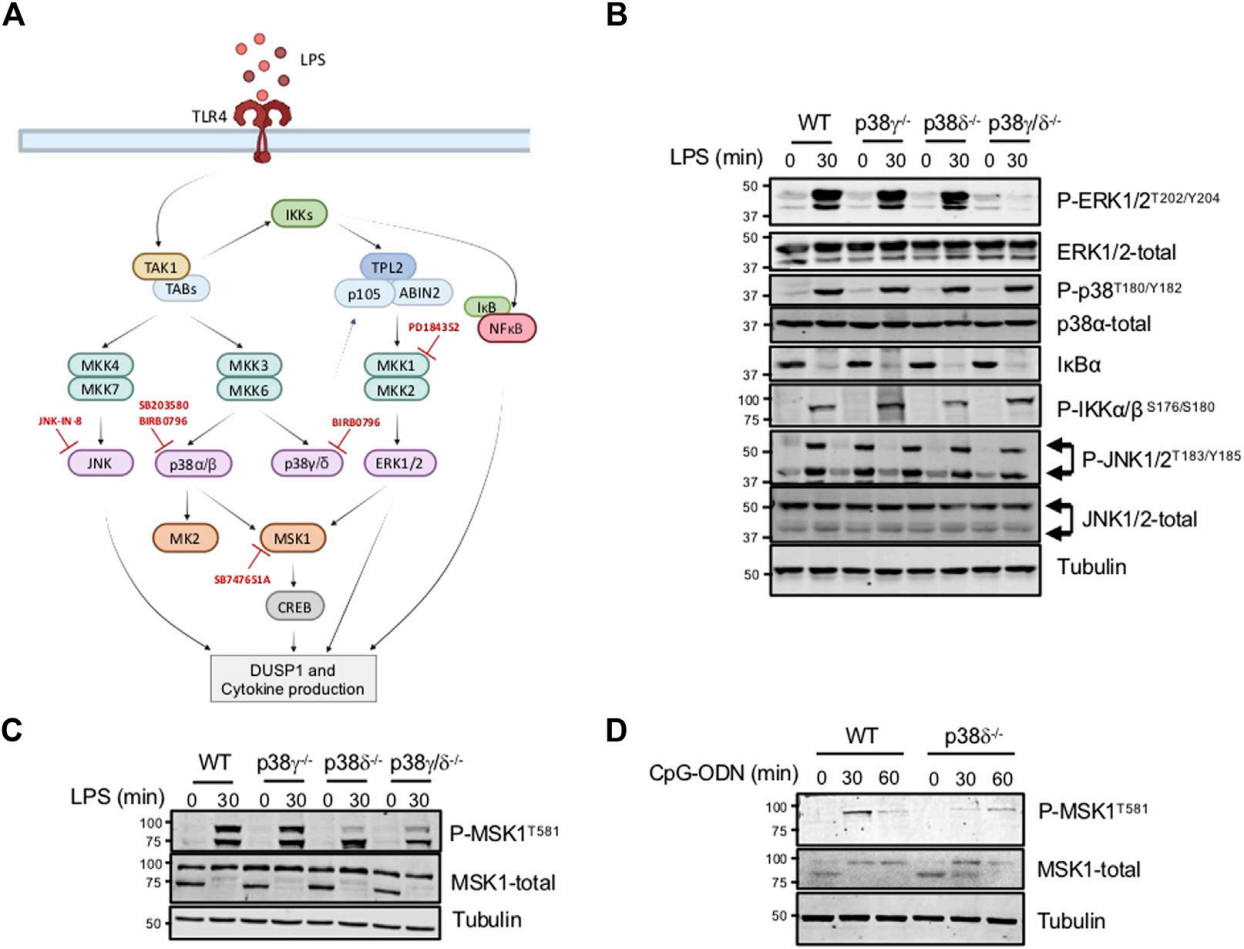
MSK1 phosphorylation in impaired in p38δ^−/−^ macrophages. **(A)** Schematic representation of the TLR4 signalling pathways involved in MAPK and NFκB pathway activation. TLR4 stimulation by LPS triggers the activation of TAK1-IKK-TPL2 *via* MyD88. p38γ and p38δ regulate TPL2 steady-state levels, which is in a complex with ABIN-2 and p105 (3, 8). The kinases blocked by the indicated inhibitors are shown. **(B,C)** BMDM from WT, p38γ^−/−^, p38δ^−/−^ or p38γ/δ^−/−^ mice were stimulated with 100 ng/mL LPS for 30 min. Cell lysates were immunoblotted with the indicated antibodies. Representative immunoblots from three independent experiments in duplicate are shown. **(D)** BMDM from WT or p38δ^−/−^ mice were stimulated with 250 ng/mL CpG-ODN for the times indicated. Cell lysates were immunoblotted with the indicated antibodies. Representative immunoblots from three different experiments in duplicate are shown. Molecular weights are indicated at the side of the blots.

**FIGURE 2:**
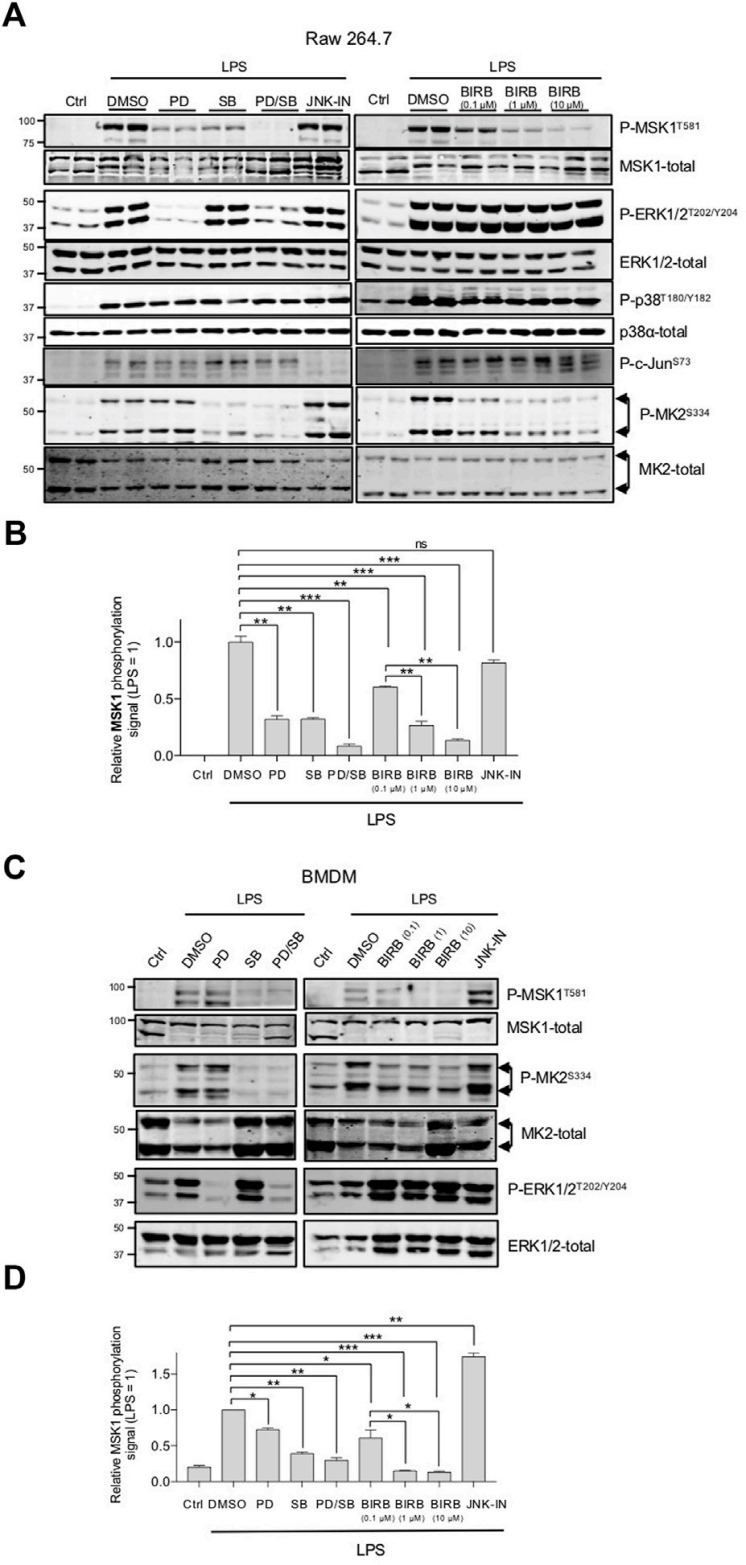
MSK1 phosphorylation in blocked by ERK1/2 and p38MAPK inhibitors in macrophages. **(A)** Raw 264.7 cells were incubated for 1 h with or without 2 µM PD184352, 10 μM SB203580, 3 µM JNK-IN-8 (to inhibit JNKs) or 0.1 µM (to inhibit p38α/β), 1 µM (to inhibit p38γ) or 10 µM (to inhibit p38δ) BIRB0796 and then stimulated with LPS as in (Figures 1B, C). Cell lysates were immunoblotted with the indicated antibodies. Representative immunoblots from two independent experiments in duplicate are shown. **(B)** MSK1 band from panel **(A)** were quantified using the Fiji program. Data show mean ± SEM from two experiments in duplicate. **p ≤ 0.01; ***p ≤ 0.001. **(C)** WT BMDM were incubated for 1 h with or without 2 µM PD184352, 10 μM SB203580, 3 µM JNK-IN-8 (to inhibit JNKs) or 0.1 µM (to inhibit p38α/β), 1 µM (to inhibit p38α/β and p38γ) or 10 µM (to inhibit p38α/β, p38γ and p38δ) BIRB0796 and then stimulated with 100 ng/mL LPS for 60 min. Cell lysates were immunoblotted with the indicated antibodies. Representative immunoblots from two independent experiments in duplicate are shown. **(D)** MSK1 band from panel **(C)** were quantified using the Fiji program. Data show mean ± SEM from two experiments in duplicate. **p ≤ 0.05; **p ≤ 0.01; ***p ≤ 0.001. In the figure, the molecular weight of the proteins is indicated at the side of the blots.

Signalling pathways activated in response to the Toll-like receptor 4 (TLR4) ligand lipopolysaccharide (LPS) (Figure 1A) were analysed in bone marrow derived macrophages (BMDM) from WT, p38γ−/−, p38δ−/− and p38γ/δ−/− mice. As reported before, the activation of ERK1/2 was impaired in p38γ/δ−/− cells, whereas p38α and JNK1/2 activation was not affected, as determined by immunoblotting with phosphospecific antibodies (Risco et al., 2012; Alsina-Beauchamp et al., 2018) (Figure 1B). IKKα/β phosphorylation and TLR4-induced NF-κB inhibitor IκBα proteolysis were also unaffected in all genotypes (Figure 1B). In contrast, the phosphorylation of the Mitogen- and Stress-activated Kinase (MSK) 1 was notably diminished in LPS stimulated p38δ−/− and p38γ/δ−/− cells (Figure 1C). MSK1 total levels were similar in all genotypes (Figure 1C). MSK1phosphorylation was also decreased in p38δ−/− BMDM stimulated with the TLR9-ligand the unmethylated CpG oligonucleotide (ODN) (Figure 1D), which shows that this effect is not restricted just to TLR4 signalling. MSK1 is activated downstream of ERK1/2 and p38α (Deak et al., 1998; Reyskens and Arthur, 2016). Consistent with this, MSK1 phosphorylation was decreased in WT LPS-stimulated Raw 264.7 macrophages and BMDM treated with the MKK1 inhibitor PD184352, to block ERK1/2 activation, or with the p38α/p38β inhibitor SB203580, to block p38α, or with both inhibitors together (Figures 2A–D). Treatment with high concentration (10 µM) of the pan-p38MAPK inhibitor BIRB0796, which at 0.1 µM inhibits p38α/p38β, at 1 µM p38γ, and at 10 µM inhibits p38δ (Kuma et al., 2005), caused a decreased in MSK1 phosphorylation larger than incubation with lower concentrations (0.1 or 1 µM) of BIRB0796 or with SB203580 alone (Figures 2A–D), supporting the idea that p38δ regulates MSK1 activation in macrophages in response to LPS. As expected, the compound PD184352 impaired ERK1/2 phosphorylation, and both SB203580 and BIRB0796 the phosphorylation of MK2, which is a p38α substrate, in LPS-stimulated macrophages (Figures 2A, C). BIRB0796 (10 µM) also inhibits JNK1/2 (Kuma et al., 2005), we then treated BMDM with the specific JNK inhibitor, JNK-IN-8 (Zhang et al., 2012), to examine if the decrease on MSK1 phosphorylation was mediated by JNK1/2 inhibition, and found that MSK1 phosphorylation was not blocked by JNK-IN-8 (Figures 2A–D), which inhibited c-Jun phosphorylation (Figure 2A).

### p38δ and MSK1phosphorylate each other *in vitro*

**FIGURE 3:**
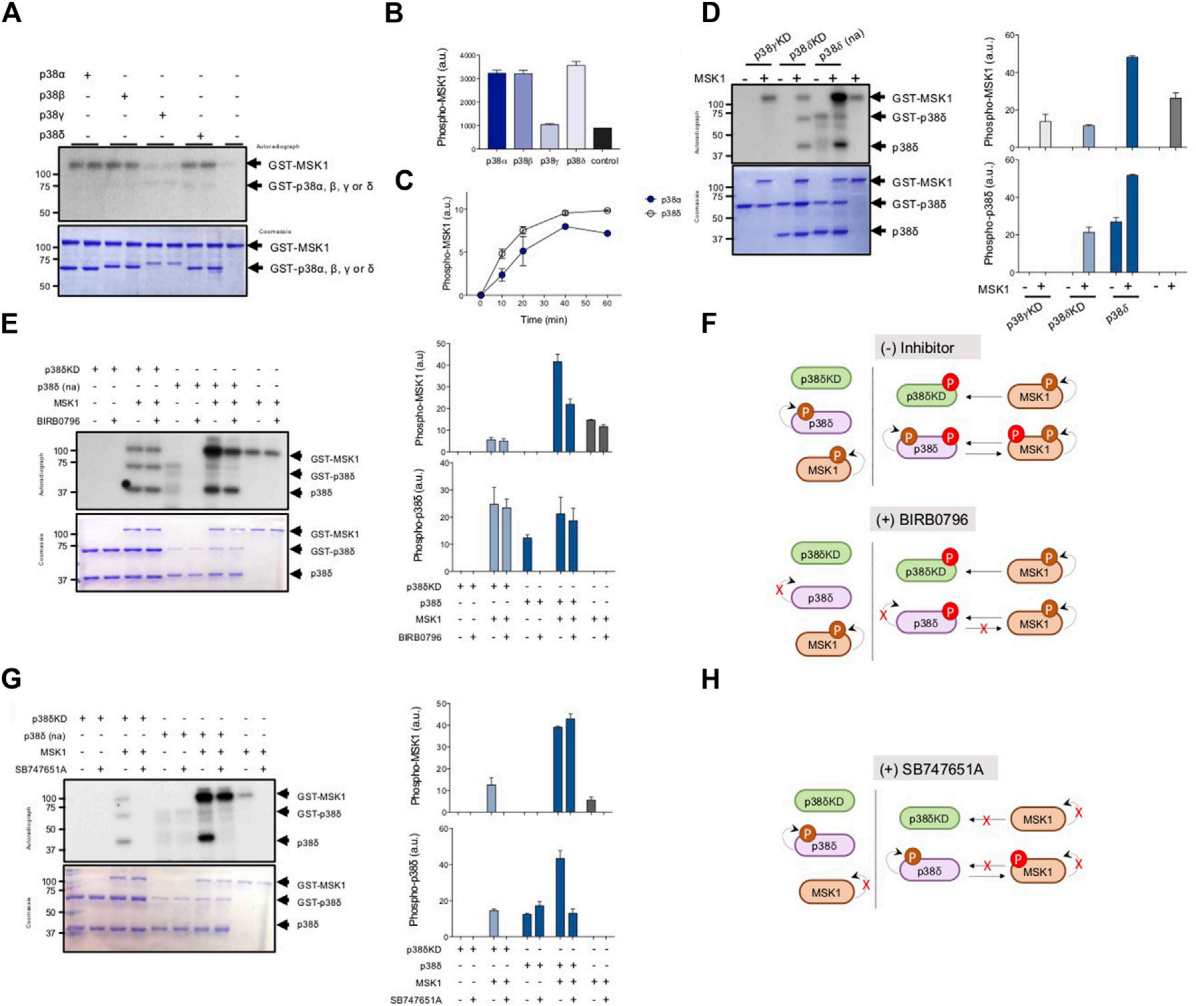
MSK1 is phosphorylated by p38δ. **(A)** Recombinant GST-MSK1 (1 µM) was incubated with active recombinant p38α, p38β, p38γ or p38δ for 60 min at 30°C in a phosphorylation reaction mix containing Mg-[γ^32^P]-ATP, as described in materials and methods. The activity of recombinant p38α, p38β, p38γ or p38δ was matched using MBP as substrate and 0.5 U/mL were used in the assay. Reaction was stopped with SDS-sample buffer. Samples were resolved in SDS-PAGE and subjected to Coomassie blue staining and autoradiography. **(B)** Bands corresponding to ^32^P-MSK1 were quantified and expressed in arbitrary units (a.u.). Data are shown as mean ± SEM from two experiments in duplicate. **(C)** Recombinant GST-MSK1 (1 μM) was incubated with p38α or p38δ (0.5 U/mL) as in **(A)** for the times indicated. Bands corresponding to ^32^P-MSK1 were quantified and data represented as mean ± SEM from two experiments in duplicate. **(D)** GST-p38γKD (1 µM), GST-p38δKD (1 µM) or GST-p38δ(na) (1 µM) were incubated with or without recombinant MSK1 (1 µM) in a phosphorylation reaction mix containing Mg-[γ^32^P]-ATP, as described in Figure 1A. ^32^P-MSK1 and ^32^P-p38 bands were quantified and data represented as mean ± SEM from two experiments in duplicate. **(E)** GST-p38δKD (1 µM) or GST-p38δ(na) (1 µM) were incubated with or without recombinant MSK1 (1 µM) and BIRB0796 (10 µM) in a phosphorylation reaction mix containing Mg-[γ^32^P]-ATP, as described in Figure 1A. ^32^P-MSK1 and ^32^P-p38δ (GST- and non-GST-tagged) bands were quantified and data represented as mean ± SEM from two experiments in duplicate (right panel). **(F)** Schematic representation of MSK1 and p38δ phosphorylation in the presence or absence of BIRB0796. **(G)** GST-p38δKD (1 µM) or GST-p38δ(na) (1 µM) were incubated with or without recombinant MSK1 (1 µM) and SB747651A (100 µM) as described in Figure 1A. ^32^P-MSK1 and ^32^P-p38δ (GST- and non-GST-tagged) bands were quantified and data represented as mean ± SEM from two experiments in duplicate (right panel). **(H)** Schematic representation of MSK1 and p38δ phosphorylation in the presence or absence of SB747651A. In the figure, the molecular weight of the proteins is indicated at the side of the blots.

MSK1 phosphorylation was reduced in p38δ−/− macrophages. Since MSK1 is an ERK1/2 and p38α substrate, but the activation of these two kinases was not affected in p38δ−/− macrophages in response to LPS, we hypothesised that p38δ could directly phosphorylate MSK1. Thus, we next examined if recombinant MSK1 was phosphorylated by active recombinant p38δ in in vitro kinase assay using Mg [γ32P]-ATP and p38α, p38β and p38γ as comparative controls. All p38MAPKs were used at the same specific activity towards myelin basic protein (MBP), which is a pan-p38MAPK substrate. GST-MSK1 was phosphorylated by p38α, p38β and p38δ, but not by p38γ (Figures 3A, B). The rate of phosphorylation of MSK1 by p38α and p38δ was similar under our experimental conditions (Figure 3C).

MSK1 autophosphorylates in multiple sites (McCoy et al., 2005), thus, there is a possibility that the presence of p38δ might be helping MSK1 autophosphorylation. To examine this, we incubated MSK1 and Mg[γ32P]-ATP, in the presence or absence of recombinant non-activated p38δ (p38δ(na)), that was not previously activated by MKK6 *in vitro*, p38δ inactive mutant (p38δD168A, a p38δ kinase dead (p38δKD)), or p38γ inactive mutant (p38γD171A; p38γKD) as control (Figure 3D). All recombinant p38δ preparations contained the GST-tagged (GST-p38s) and the non-GST-tagged (p38s) protein, probably due to the cleavage of the GST part after purification (Figure 3D). We found similar MSK1 autophosphorylation in the absence of p38 and in the presence of p38δKD and p38γKD; however, MSK1 autophosphorylation/phosphorylation was increased in the presence of p38δ (Figure 3D). In addition, we observed autophosphorylation of the wild type p38δ(na), but not p38δKD or p38γKD (Figure 3D). These results indicate that basal kinase activity of recombinant p38δ could account for the increase in MSK1 phosphorylation (Figure 3D). Additionally, both p38δ and p38δKD phosphorylation was significantly increased in the presence of MSK1 (Figure 3D), suggesting that p38δ is directly phosphorylated by MSK1.

To check if MSK1 and p38δ phosphorylate each other we studied their phosphorylation in the presence or absence of 10 µM BIRB0796 to inhibit p38δ, or 100 µM SB747651A to inhibit MSK1 (Kuma et al., 2005; Naqvi et al., 2012). As expected, incubation with the BIRB0796 inhibitor blocked p38δ autophosphorylation and decreased MSK1 phosphorylation by p38δ to similar levels to that of MSK1 autophosphorylation (Figure 3E). BIRB0796 did not impaired either p38δKD or p38δ phosphorylation in the presence of MSK1 (Figure 3E). These data confirmed that p38δ directly phosphorylates MSK1 and strongly suggest that MSK1 directly phosphorylates p38δ (Figure 3F). Incubation with SB747651A blocked MSK1 autophosphorylation, but did not inhibit the phosphorylation of MSK1 by p38δ (Figure 3G). However, SB747651A impaired p38δKD and p38δ phosphorylation by MSK1, but not p38δ autophosphorylation (Figure 3G), showing that MSK1 directly phosphorylates p38δ (Figure 3H).

### p38δ activates MSK1

**FIGURE 4:**
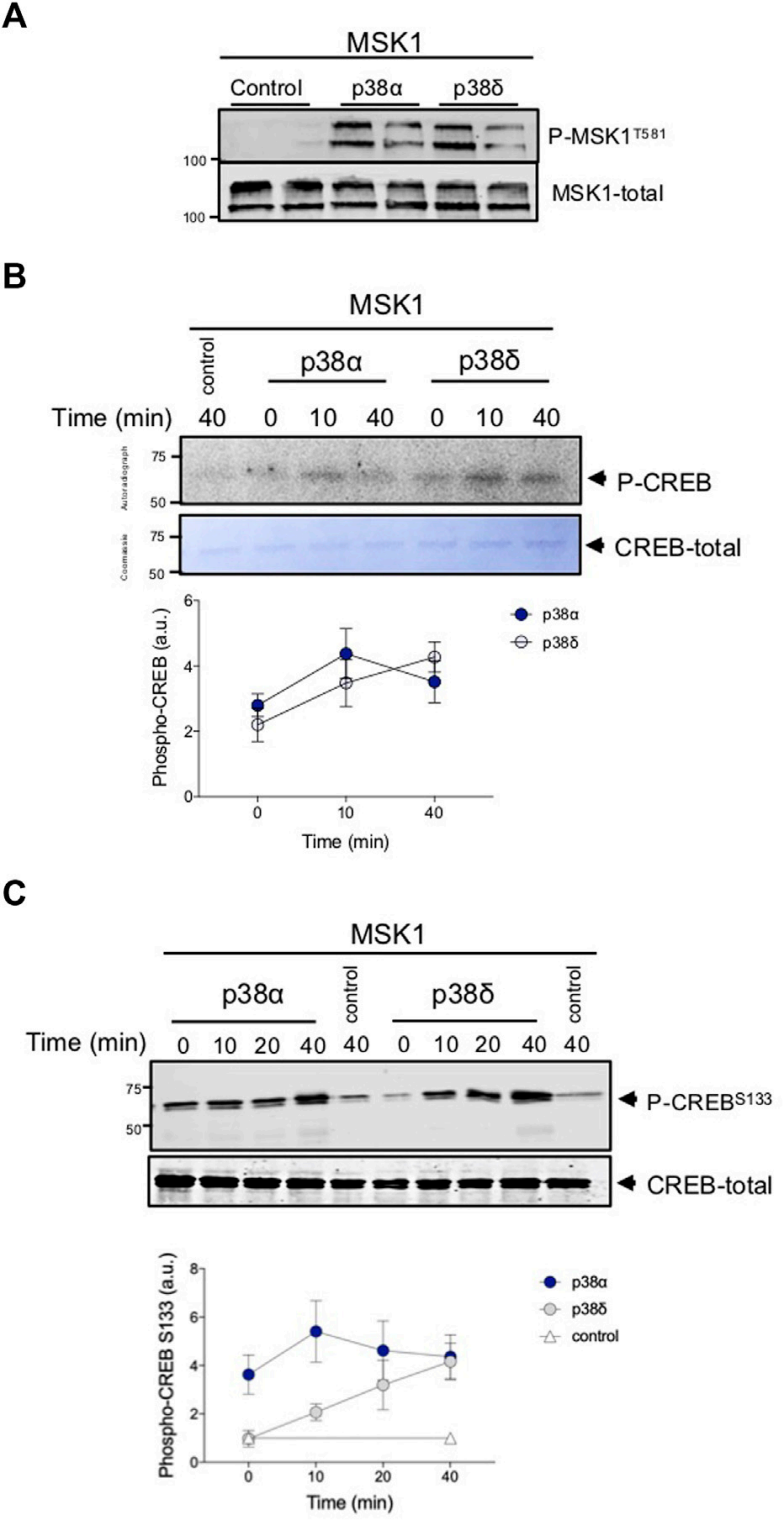
MSK1 is activated by p38δ. **(A)** Recombinant GST-MSK1 (100 ng) was incubated with active recombinant p38δ (0.5 U/mL) for 60 min at 30°C in a phosphorylation reaction mix as described in materials and methods. Reaction was stopped with SDS-sample buffer and samples immunoblotted with the indicated antibodies. MSK1 phosphorylation was detected using the Phospho-MSK1 (Thr581) antibody. Representative immunoblots from two independent experiments in duplicate are shown. **(B)** Recombinant CREB (1 µg) was incubated with active MSK1 as described in materials and methods. Reaction was stopped with SDS-sample buffer. Samples were resolved in SDS-PAGE and subjected to Coomassie blue staining and autoradiography. Phospho-CREB bands were quantified and data represented in arbitrary units (a.u.) as mean ± SEM from two experiments in duplicate (lower panel). **(C)** Recombinant GST-CREB (50 ng) was incubated with active recombinant MSK1 as in panel **(B)** for the indicated times at 30°C in a phosphorylation reaction mix as described in materials and methods. Reaction was stopped with SDS-sample buffer and samples immunoblotted with the Phospho-CREB (Ser133) antibody. Representative immunoblots from two independent experiments are shown. Phospho-CREB bands were quantified and data represented as mean ± SEM from three experiments in duplicate (lower panel). The molecular weight of the proteins is indicated at the side of the blots.

*In vitro* MSK1 phosphorylation by p38δ was confirmed by western blot using the antibody against Phospho-T581 (Figure 4A), which is the proline-direct phosphorylation site essential for MSK1 activation directly phosphorylated by ERK1/2 and p38α (McCoy et al., 2005). We then study whether or not phosphorylation by p38δ activates MSK1 *in vitro*. For this, we use the transcription factor CREB as MSK1 substrate. CREB is a MSK1 physiological substrate (Reyskens and Arthur, 2016). We found that the incubation with Mg[γ32P]-ATP and activated p38δ or p38α enhanced CREB phosphorylation. (Figure 4B). As a positive control, we confirmed that MSK1 was activated with active p38α. The ability of p38δ or p38α to activate GST-MSK1 correlated with the extent of phosphorylation of this kinase (Figure 3C). MSK1 activation by p38δ was confirmed analysing CREB phosphorylation by immunoblot, using the anti-Phospho-CREB (S133) antibody (Figure 4C). This antibody recognized the CREB residue (S133) specifically phosphorylated by MSK1 (Deak et al., 1998). These data show that MSK1 is phosphorylated and activated by p38δ *in vitro*.

### p38δ deletion impairs the phosphorylation of MSK1 downstream targets in BMDM

**FIGURE 5:**
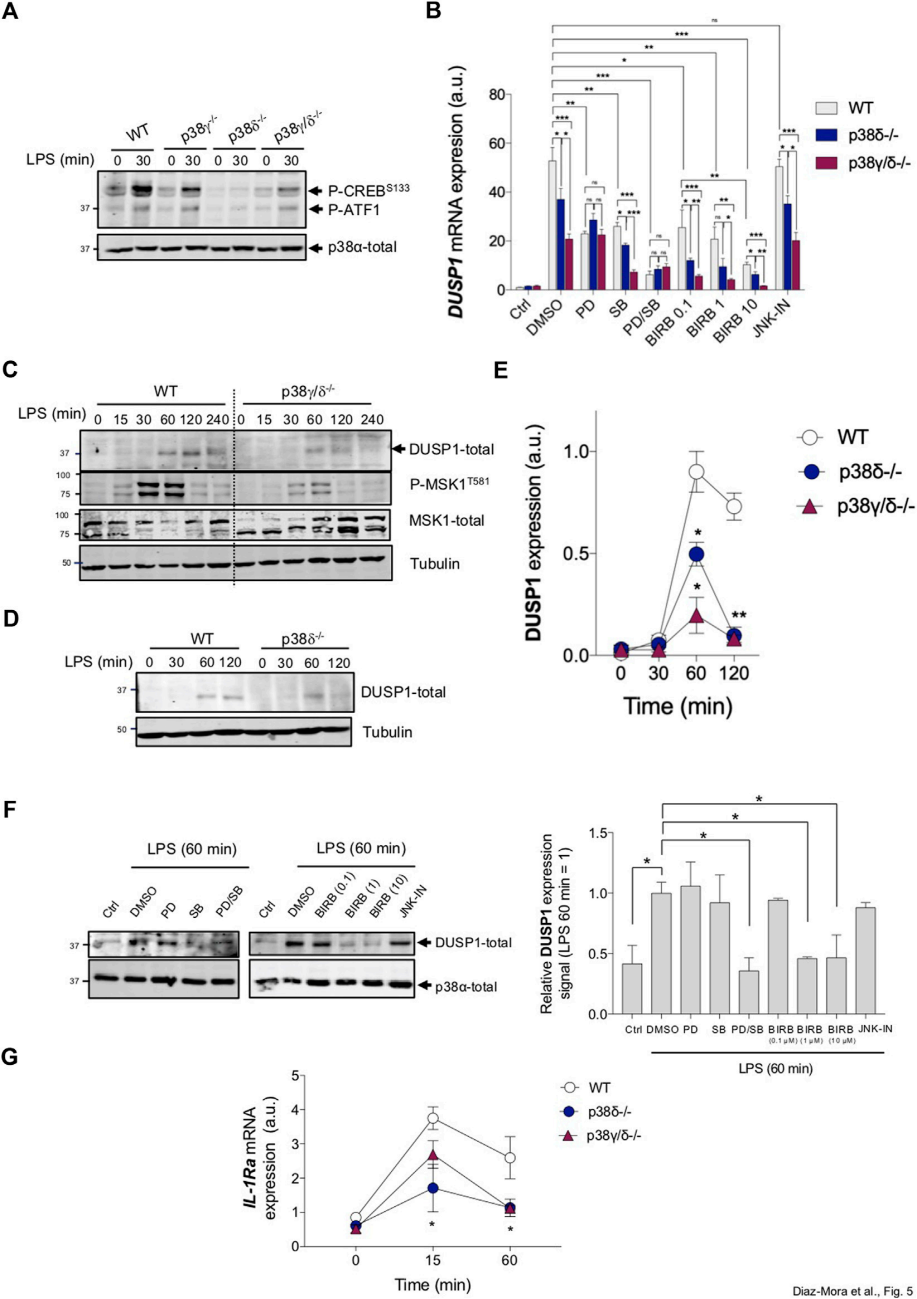
Deletion of p38δ impairs the activation of MSK1 downstream targets. **(A)** BMDM from WT, p38γ^−/−^, p38δ^−/−^ or p38γ/δ^−/−^ mice were stimulated with 100 ng/mL LPS for 30 min. Cell lysates were immunoblotted with the indicated antibodies. Phospho-CREB (Ser133) antibody also recognises phosphorylated ATF1. Representative immunoblots from three independent experiments in duplicate are shown. **(B)** WT, p38δ^−/−^ or p38γ/δ^−/−^ BMDM were exposed to 100 ng/mL LPS for 1 h with or without 2 µM PD184352, 10 μM SB203580, 3 µM JNK-IN-8 (to inhibit JNKs) or 0.1 µM (to inhibit p38α/β), 1 µM (to inhibit p38α/β and p38γ) or 10 µM (to inhibit p38α/β, p38γ and p38δ) BIRB0796 and then stimulated with LPS. Relative mRNA expression was determined by qPCR for DUSP1. Results were normalized to β-actin mRNA expression and x-fold induction was calculated relative to WT expression at 0 h control. Data show mean ± SEM from one representative experiment of three in triplicate, with similar results. *p ≤ 0.05, **p ≤ 0.001. **(C,D)** BMDM from WT, p38γ/δ−/− or p38δ−/− mice were stimulated with 100 ng/mL LPS for the indicated times. Cell lysates were immunoblotted with the indicated antibodies. Representative immunoblots from three independent experiments in duplicate are shown. **(E)** DUSP1 bands from blots shown in panels **(C,D)** were quantified and data represented in arbitrary units (a.u.) as mean ± SEM from three experiments in duplicate. *p ≤ 0.05; **p ≤ 0.001 relative to WT. **(F)** WT BMDM were incubated for 1 h with or without 2 µM PD184352, 10 μM SB203580, 3 µM JNK-IN-8 or 0.1, 1 or 10 µM BIRB0796 and then stimulated with LPS. Cell lysates were immunoblotted with the indicated antibodies. Representative immunoblots from two independent experiments in duplicate are shown. DUSP1 bands were quantified and data represented as mean ± SEM from two experiments in duplicate (lower panels). Molecular weight of the proteins is indicated at the side of the blots **(G)** BMDM were exposed for different times to 100 ng/mL LPS. Relative mRNA expression was determined by qPCR for *IL-1Ra*. Results were normalized to *β-actin* mRNA expression and x-fold induction was calculated relative to WT expression at 0 h. Data show mean ± SEM from one representative experiment of two in triplicate, with similar results. *p ≤ 0.05.

We then evaluated the role of p38δ in mediating the phosphorylation of CREB at S133, and also of its close relative transcription factor ATF1 at the equivalent residue, S63, in cells. We found that CREB and ATF1 are phosphorylated after treatment of WT and p38γ−/− BMDM with LPS (Figure 5A). In contrast, the lack of p38δ significantly decreased CREB and ATF1 phosphorylation in LPS-stimulated p38δ−/− BMDM, and also in p38γ/δ−/− BMDM, although to a less extent (Figure 5A).

One role of MSK in macrophages is to regulate the expression of immediate early genes, such as dual specificity protein phosphatase 1 (DUSP1), through CREB phosphorylation (Arthur et al., 2004; Ananieva et al., 2008). We found that LPS-induced DUSP1 mRNA expression was significantly decreased in p38δ−/− and p38γ/δ−/− BMDM compared to WT cells (Figure 5B). In all WT, p38δ−/− and p38γ/δ−/− macrophages, DUSP1 mRNA expression, in response to LPS, was affected in the presence of p38 inhibitors, SB203580 or BIRB0796, and this effect was more pronounced in p38γ/δ−/− cells and at high concentration of BIRB0796 (Figure 5B). LPS stimulation also induced DUSP1 protein expression in WT macrophages, which was significantly impaired in p38γ/δ−/− and p38δ−/− BMDM (Figures 5C–E). Consistent with this finding, in WT macrophages, the expression of DUSP1 protein induced by LPS was blocked by preincubation with high concentrations of BIRB0796 to levels comparative to those observed after preincubation with a combination of both PD184352 and SB203580 (Figure 5F). Preincubation with PD184352 or SB203580 alone, or with JNK-IN-8 did not affect DUSP1 protein expression (Figure 5F). All these results indicate that p38δ is a key player in the regulation of DUSP1 expression in response to TLR4 activation in macrophages.

MSKs also regulate the transcription of the anti-inflammatory molecule, the IL-1 receptor antagonist (IL-1Ra) in macrophages (Darragh et al., 2010). Consistent with the involvement of p38δ in controlling MSK activation, IL-1Ra mRNA expression was significantly blocked in LPS-stimulated p38δ−/− and p38γ/δ−/− BMDM, compared to WT cells (Figure 5G). All these data indicate that p38δ positively regulates anti-inflammatory signalling.

## Discussion

We provide evidence that MSK phosphorylation is mediated by p38δ *in vitro* and in macrophages and, on the basis of this, propose a new way of MSK activation in cells. It is well stablished that MSKs are regulated by multiple phosphorylation. Both p38α and ERK1/2 mediate MSK phosphorylation at T581 and S360 in cells. Of these, T581 is required for the activation of MSK C-terminal kinase domain, in response to cellular stress or mitogens, and causes the autophosphorylation in other MSK domains (Deak et al., 1998; McCoy et al., 2005; Reyskens and Arthur, 2016). We found that p38δ phosphorylates MSK1 at T581, which causes the MSK1 activation *in vitro*. Interestingly, we also observed that MSK1 phosphorylates p38δ in in vitro experiments. MSK1 might be phosphorylating p38δ at S361, which is at the C-terminus end of p38δ, since it lies in a MSK phosphorylation consensus motif RRXS. This S361 residue is not conserved in either p38γ or other p38MAPKs. Non-etheless, the identification of p38δ residues that are phosphorylated by MSK1 remains to be elucidated. Further experiments would be also required to study whether MSK1 phosphorylates p38δ and regulates its function in cells.

The specific phosphorylation of T581 is widely used as a read out of MSK activation in cells. This phosphorylation is severely reduced in LPS-stimulated macrophages from p38δ- or p38γ/δ-null mice, as well as in macrophages stimulated with LPS in the presence of high concentrations of the pan-p38MAPK inhibitor BIRB0796, supporting that p38δ regulates MSK activation by direct phosphorylation in response to LPS in macrophages. Consequently, either p38δ inhibition or deletion leads to a blockade of CREB and ATF1 phosphorylation and of the expression of CREB-dependent genes encoding anti-inflammatory proteins DUSP1 and IL-1Ra. Surprisingly, MSK1, CREB and ATF1 phosphorylation in p38γ/δ−/− macrophages seems to be higher than in p38δ−/− cells; this could be due to the functional redundancy between related family members. For example, it has been shown that the protein hDlg is a p38γ physiological substrate in mouse embryonic fibroblasts (MEF); however, this is phosphorylated by p38δ in p38γ−/− MEFs, and by p38α in p38γ/δ−/− cells (Escós et al., 2016; Cuenda and Sanz-Ezquerro, 2017). Thus, there is the possibility that p38α can be phosphorylating MSK1, CREB and ATF1 in p38γ/δ−/−, but not in p38δ−/− macrophages.

DUSP1 is a dual specificity phosphatase that inactivates p38α and JNK1/2 (Keyse, 2008). In LPS-stimulated macrophages, p38α and JNK1/2 are transiently phosphorylated/activated, reaching their maximal phosphorylation between 15–30 min, and being dephosphorylated after that time (Risco et al., 2012; Alsina-Beauchamp et al., 2018). In the case of JNK1/2, but not p38α, the dephosphorylation phase is significantly slower in p38γ/δ−/− than in WT BMDM (Risco et al., 2012). This delayed JNK1/2 dephosphorylation might be due to the low expression of DUSP1 in p38γ/δ−/− macrophages, and suggest that another phosphatase may be dephosphorylating p38α in those cells.

In addition to DUSP1 and IL-1Ra, MSKs regulate the expression of the anti-inflammatory cytokine IL-10 in macrophages. It has been shown that MSK-mediated phosphorylation of CREB at Ser133 is required for its binding to the promoter of IL-10 after LPS stimulation in BMDM. IL-10 production is inhibited in MSK1/2−/− BMDM compared to WT (Ananieva et al., 2008). Accordingly, we have previously described that IL-10 transcription is partially blocked in p38δ−/− and p38γ/δ−/− macrophages in response to LPS or C. albicans infection (Risco et al., 2012; Alsina-Beauchamp et al., 2018). Although deeper analyses are required, all these results point out that p38δ can activate anti-inflammatory pathways, through the activation of MSKs downstream of TLRs, that are critical for preventing uncontrolled inflammation. This is supported by our observations in colitis and in colorectal cancer (CRC) patients, where there is a negative correlation between the levels of p38δ and inflammation (Fajardo et al., 2022). The levels of p38δ are significantly decreased in samples from colitis and CRC patients, compared with samples from healthy donors (Fajardo et al., 2022).

In summary, we show here that, even in the presence of fully active p38α and ERK1/2, p38δ is essential for MSK1 phosphorylation/activation in macrophages. Also, p38δ regulates MSK1 downstream targets and this could limit the inflammatory signalling pathway downstream of TLRs.

## Data Availability

The original contributions presented in the study are included in the article/supplementary material, further inquiries can be directed to the corresponding author.
